# Case of Thyrotoxic Periodic Paralysis in a Caucasian Male and Review of Literature

**DOI:** 10.1155/2014/314262

**Published:** 2014-11-17

**Authors:** Tina K. Thethi, Rosemarie Parks, Bonnie Katalenich, Pankdeep Chhabra, Julie McCaw, Stephanie Syu, Tuyen Nguyen, Joshua Larrazolo, Kartik Munshi, Jay Waddadar, Prathima Nagireddy

**Affiliations:** ^1^Section of Endocrinology, Tulane University Health Sciences Center, 1430 Tulane Avenue, SL-53, New Orleans, LA 70112, USA; ^2^Southeast Louisiana Veterans Health Care Systems, New Orleans, LA 70112, USA; ^3^Southeast Georgia Department of Health, Waycross, LA 31501, USA; ^4^Tulane University Health Sciences Center, New Orleans, LA 70112, USA; ^5^Sanofi Pasteur India Ltd, Mumbai 400093, India; ^6^University of New Orleans, New Orleans, LA 70148, USA; ^7^St. Joseph Mercy Oakland, Pontiac, MI 48341, USA

## Abstract

*Objective*. Thyrotoxic periodic paralysis (TPP), a known condition in Asian men, is becoming increasingly common in men from Western countries. Since suspicion for TPP as a differential in diagnosis is of utmost importance to avoid overcorrection of hypokalemia and other complications, we are reporting a case of TPP in a 25-year-old Caucasian male. *Methods*. The patient presented with intermittent lower extremity weakness after consumption of a large high-carbohydrate meal. Clinical examination revealed diffusely enlarged thyroid gland, no muscle power in lower extremities, tremors, and brisk deep tendon reflexes. *Results*. Clinical and laboratory findings were consistent with Graves' disease and the patient had hypokalemia. The patient responded to potassium repletion and was treated with propylthiouracil and propranolol. After treatment with radioactive iodine, the patient developed postablative hypothyroidism for which he was treated with levothyroxine. *Conclusion*. Since this condition is overlooked by physicians in Western countries, we present a case of TPP in a Caucasian male thus showing the importance of consideration of TPP in Caucasians despite its rare occurrence and the need for prompt diagnosis to avoid the danger of hyperkalemia in management of the paralytic attack in TPP patients.

## 1. Introduction

Thyrotoxic periodic paralysis (TPP), a rare condition in Western countries, is characterized by elevated thyroid hormone, hypokalemia, and muscle paralysis. Physicians in the emergency department (ED) should consider TPP in patients with these symptoms, since early diagnosis is critical to avoid overcorrection of potassium and cardiovascular and respiratory complications.

## 2. Patient Report

A 25-year-old Caucasian male was brought to ED with sudden onset weakness in the proximal lower extremity muscles. The patient reported no history of trauma. Vision and mental status were normal. Further questioning revealed that the patient ate a large portion of pasta several hours prior. The patient also reported a history of less severe episodes of weakness over several months, usually on weekend nights, sometimes after ingestion of alcohol. Symptoms resolved spontaneously within hours. Family history was unobtainable as patient was adopted. On presentation, heart rate was 117/min and blood pressure (BP) was 176/85 mm/Hg.

Examination revealed no proptosis, diffusely enlarged thyroid gland, without bruit or nodules. His muscle strength was 0/5 in lower extremity proximal muscles, with no sensory deficits, with upper extremities being 5/5 bilaterally. There was no impairment of cognitive or cerebellar function. His hands exhibited tremors, and deep tendon reflexes (DTRs) were brisk bilaterally without fasciculations or clonus. Remainder of the examination was normal.

Laboratory data showed abnormal results for creatinine kinase, 531 U/L (normal range 38–174 U/L) and serum potassium, 1.7 mEq/L (normal range 3.5–5 mEq/L). Electrocardiogram revealed sinus tachycardia with left ventricular hypertrophy. He had biochemical evidence of hyperthyroidism with the following values: total serum thyroxine (T4) = 18.4 mcg/dL (normal range 5 to 12 mcg/dL), free T4 = 4.2 ng/dL (normal range 0.7 to 1.9 ng/dL), and T3 = 255 ng/dL (normal range 60−180 ng/dL). TSH was <0.005 (normal range 0.4 to 4.5 microU/mL). Urinary potassium was normal. Thyroid uptake and scan showed radioactive iodine uptake consistent with Graves' disease (48.2% at 2 hours and 66% at 24 hours).

In ED, the patient was treated with hydration and potassium supplementation. Potassium returned to normal (3.5–5 mEq/L) with infusion of 40 mEq. Eight hours later, the patient regained total lower body strength. He was started on propylthiouracil and propranolol and scheduled for outpatient follow-up. Propylthiouracil was chosen as it was covered by his insurance plan. He developed postablative hypothyroidism after being treated with I131 12 millicuries for definitive management and was started on thyroid replacement therapy. He remained euthyroid for six months after which he was lost to follow up.

## 3. Discussion

### 3.1. Epidemiology of TPP

Hypokalemic paralysis can occur as a deficiency due to excess loss of potassium, or in a periodic form caused by intracellular movement of potassium (hypokalemic periodic paralysis (HPP)). HPP is an unusual metabolic disorder which occurs in association with thyrotoxicosis or, more commonly, in familial form (familial hypokalemic periodic paralysis (FHPP)). FHPP is most frequent in Western countries while TPP is most common in Asian populations, [1]. In a retrospective analysis, patients of Polynesian ethnicity (Maori and Pacific Islander), 71% of the 21 patients with TPP had HPP [2], thus making them an ethnic group at an increased risk for TPP in addition to the Asians. Incidence of TPP in American Caucasians is as low as 0.1-0.2% among those patients with hyperthyroidism [3].

The potential causes of thyrotoxicosis which have been associated with TPP are Graves' disease, toxic multinodular goiter, solitary toxic adenoma, de Quervain's thyroiditis, thyroid hormone overdose, TSH-secreting pituitary adenoma, Jod-Basedow disease, amiodarone therapy, and triiodothyronine-containing weight-reducing agents [4]. Despite a higher incidence of thyrotoxicosis in females, cases of TPP have a male to female ratio of 20 : 1 [1].

The age of onset of TPP is usually 20–40 years, which coincides with commonly observed age distribution for hyperthyroidism [4, 5].  Table 1 shows the distinguishing features between TPP and FHPP. Similarities include presence of hypokalemia, clinical signs, and use of potassium salts to abort attack and prevention of further attacks by propranolol. There are no histological differences in muscle biopsy.

### 3.2. Pathophysiology

Pathogenesis of TPP may be related to changes in activity of Na^+^/K^+^ channels in thyrotoxicosis. Thyroxine may augment cell membrane Na^+^/K^+^ATPase activity causing potassium influx into the cell [6–8]. In periodic paralysis, Na^+^/K^+^ATPase activity is increased to a much greater extent than in other thyrotoxic subjects due to higher sensitivity to thyroid hormones. Hyperthyroidism is a hyper adrenergic state in which beta-2-adrenergic stimulation in muscle cells directly induces cellular K^+^ uptake by increasing cAMP, leading to activation of Na^+^/K^+^ATPase [9, 10]. Thyroid hormone also directly stimulates Na^+^/K^+^ATPase and increases the number and sensitivity of beta-receptors [6, 11, 12]. This increase in Na^+^/K^+^ATPase activity can lead to a shift of potassium ions from extracellular to intracellular compartments. Thus hypokalemia is due to the intracellular shift of potassium, not potassium depletion [12], so only a small amount of potassium is required to correct it. Total body potassium, urinary potassium, and fecal potassium excretion are normal.

Diurnal variation in potassium metabolism (i.e., nighttime influx of potassium into skeletal muscle and daytime efflux from intracellular compartment to extracellular fluid) may explain why paralytic attacks occur at night [13]. However hypokalemia cannot be solely responsible for neuromuscular manifestations of TPP as many euthyroid cases have hypokalemia but no paralysis, while in some cases there is recovery during hypokalemia. Insulin also may activate Na^+^/K^+^ATPase activity, which explains the correlation between periodic paralysis and high-carbohydrate meals. Whether TPP patients have a genetic predisposition for activation of the Na^+^/K^+^ATPase genes is unknown.

During the paralysis phase, there is a decrease in both duration of CMAP (compound muscle action potential) and amplitude of evoked muscle action potential on nerve stimulation, while polyphasic potential is increased [14]. Electron microscopy indicates that the presence of vacuolation is a hallmark of TPP, irrespective of potassium level and muscle weakness [15].

**Figure 1:**
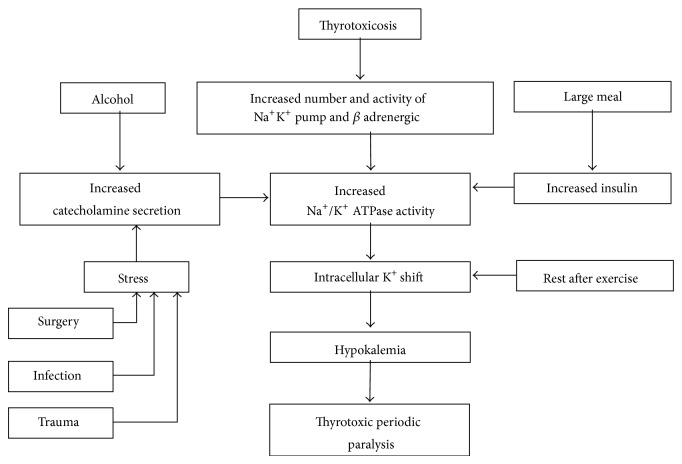
Precipitants of periodic paralysis.

Muscle biopsy findings may be similar to those in FHPP. Other precipitants of periodic paralysis are shown in Figure 1.

### 3.3. Clinical Features

#### 3.3.1. Thyrotoxic Features

Clinical features of thyrotoxic state may be subtle in TPP. Clues to the diagnosis include warm moist skin, fever, and tachycardia with widened pulse pressure, exophthalmos, and goiter.

Elevated pulse and BP are more sensitive indicators of TPP than other classic signs of hyperthyroidism.

#### 3.3.2. Neuromuscular Features

Typically, paralysis is in the lower limbs. Bilateral weakness affecting proximal muscles is suggestive of TPP. Patients usually present to ED at night or early morning, as the weakness typically starts after dinner and rest. Weakness can also result from various psychophysical stresses, alcohol consumption, strenuous exercise or drugs like *β*2-adrenergic agonists. Paralysis usually resolves within 1–36 hours, in the reverse order of appearance, and may be hastened by administration of potassium. There is no involvement of the sensory system, cranial nerves, bulbar or ocular muscles. Rarely, TPP can affect respiratory muscles with the potential for severe and even fatal complications [16]. DTRs are weak or absent, but anal sphincter tone and urinary continence are preserved. The clinical features of TPP do not differ from FHPP except for the hyperthyroidism present in TPP. These findings differentiate TPP from Guillain-Barre syndrome, myasthenia gravis, botulism, and transverse myelitis.

#### 3.3.3. Biochemical Changes

The main feature of TPP is hypokalemia due to intracellular shift of K^+^ ions and not actual depletion. Hypokalemia is the most consistent electrolyte abnormality and a hallmark of TPP, along with hyperthyroidism. It is important to administer small doses of potassium with frequent monitoring of serum potassium, as potassium which entered the cell during paralysis is also released, further raising the serum potassium. One study showed that potassium supplementation resulted in rebound hyperkalemia in over 40% of cases [12]. Hypophosphatemia during paralysis in patients with TPP has been reported and may contribute to muscle weakness. Hypophosphatemia and hypomagnesaemia can be attributed to intracellular movement induced by catecholamines and return to normal without supplementation once the patient recovers from paralysis.

Because thyroid hormones affect bone, urinary phosphate excretion is reduced and calcium excretion is increased. In the ED, a spot urine sample showing a calcium/phosphate ratio of 1.7 or higher can provide an early diagnosis of TPP. This test has a predictive value with sensitivity of 100% and specificity of 96% [17].

#### 3.3.4. Electrocardiogram Changes

Triad of prolonged QT-U interval, prolonged PR interval, and a resting sinus tachycardia are the significant ECG changes seen in TPP. Correcting hypokalemia and paralysis with excess intravenous potassium, dextrose infusion, and beta adrenergic stimulation (e.g., adrenaline administration) may cause a further drop in potassium and precipitate ventricular arrhythmias [18].

#### 3.3.5. Treatment

The immediate focus of treatment should be reversal of paralysis, monitoring and treating respiratory and/or cardiac complications, and correcting hypokalemia. Risk of rebound hyperkalemia raises doubts whether potassium supplementation is needed to reverse paralysis.

Manoukain et al. [12] reported that rebound hyperkalemia (>5.0 mmol/L) occurred in nearly 40% of patients with TPP, especially if more than 90 mmol KCl was given within 24 hours. Since propranolol reverses the adrenergic overstimulation of Na^+^/K^+^ATPase and blocks the intracellular sequestration of potassium, paralytic symptoms of TPP may be rapidly terminated with high-dose oral propranolol (3-4 mg/kg) [19–21].

The cause of hyperthyroidism should be identified and definitive treatment instituted while avoiding precipitating factors of paralysis. Propranolol along with an antithyroid agent helps prevent relapse of paralysis. However, the dose of propranolol must be adequate as cases of TPP have been reported while patients are receiving low dose propranolol.

## 4. Conclusion

We present a case of TPP in a Caucasian male with typical symptoms, signs, and workup for Graves' disease with response to potassium, propranolol, and propylthiouracil therapy. TPP should be considered in the differential diagnosis for patients with similar presentations.

## Figures and Tables

**Table 1:** Differences between thyrotoxic periodic paralysis (TPP) and familial hypokalemic periodic paralysis (FHPP).

Characteristic	TPP	FHPP
Age	20–40 years	Usually less than 20 years
Heredity	Sporadic	Autosomal dominant
Ethnicity	Predominantly Asian	Predominantly Caucasians
Sex	Males more than females	Not specific
Hyperthyroidism	Present	Not present
Family history	Thyrotoxicosis	Hypokalemic paralysis
Genetic predisposition	Associated with SNPs of CACN1AS^*^ in Asians	CACN1AS^*^, KCNE3^*^, and SCN4A^*^ genes
Response to epinephrine	No change	Electromyography shows marked decrease in compound muscle action potential amplitude

^*^SNPs = single nucleotide polymorphisms.

^*^CACN1AS = calcium channel alpha-1 subunit.

^*^KCNE3 = potassium channel voltage-gated, Isk-related family, member 3.

^*^SCN4A = sodium channel alpha-subunit.
